# Clinicopathological Patterns of Malignant Solid Tumors in Adult Patients: A Hospital-Based Study From Bangladesh

**DOI:** 10.7759/cureus.34925

**Published:** 2023-02-13

**Authors:** Ayesha Siddika, Sawrab Chowdhury, Md Rockyb Hasan, Md Moniruzzaman, S.K. Jakaria Been Sayeed, Tahsin Tabassum, Mahidul Chowduary, Tanzin Tabassum, Azizul Islam, Md. Mujibur Rahman

**Affiliations:** 1 Cardiology, National Heart Foundation Hospital & Research Institute, Dhaka, BGD; 2 Computer Science and Electrical Engineering, Chandpur Polytechnic Institute, Chandpur, BGD; 3 Internal Medicine, Texas Tech University Health Sciences Center, Amarillo, USA; 4 Clinical Neurology, National Institute of Neurosciences & Hospital, Dhaka, BGD; 5 Medicine and Rheumatology, National Institute of Neurosciences & Hospital, Dhaka, BGD; 6 School of Community Health and Policy, Morgan State University, Baltimore, USA; 7 Internal Medicine, Interfaith Medical Center, Brooklyn, USA; 8 General Surgery, Lister Hospital, Stevenage, GBR; 9 Internal Medicine, Bangladesh Armed Forces, Directorate General of Medical Service (DGMS), Dhaka, BGD; 10 Medicine, Bangabandhu Sheikh Mujib Medical University, Dhaka, BGD

**Keywords:** solid tumor, risk factors, pathological pattern, clinical features, socio-economic groups, malignant solid tumor

## Abstract

Background: Solid malignant tumors are abnormal masses of tissue that usually do not contain any cysts or liquid areas. The causation of these tumors is multifactorial, and the disease process differs at different sites.

Aim: This study aims to determine the clinicopathological patterns of malignant solid tumors in adult patients admitted into the department of internal medicine of a tertiary care hospital in Bangladesh.

Method: This cross-sectional study was conducted between January 2018 and June 2018 at the Department of Medicine, Dhaka Medical College Hospital, Bangladesh. We recorded the complete socio-demographic characteristics, clinical patterns, and pathological characteristics of malignant solid tumors in adult patients.

Results: A total of 100 patients with confirmed malignant solid tumors were included in our study. The mean age of the patients was 47.5 years (SD: ±4.20); most of them (27%) were between 58 and 67 years of age. Male patients constitute 59% of the total study sample. Most of the patients were of the middle socio-economic class (59%) and most of them (61%) were nonsmokers. Among the patient diagnoses, 19% had lung cancer, 29.27% had breast cancer, and in 14%, lymphoma (tumor arising from the lymphatic system) was the common solid tumor. Lung cancer was found to be the most common form of cancer in males. Additionally, the majority of those diagnosed with lung cancer smoked. Breast cancer was found to be the most common type of cancer in females.

Conclusion: This study reflects that solid malignant tumors can affect any socio-economic class of people. Based on findings in our study as well as others, prevention efforts should focus on the reduction in tobacco use and the incorporation of other lifestyle changes, such as diet modification and exercise. Additionally, the incorporation of economic factors and how they affect cancer presentation in different contexts is crucial.

## Introduction

Cancers originate from cells almost anywhere in the human body and can grow uncontrollably. If the spread of cancers is not controlled, it can often result in death. Although the etiology of different types of cancers remains unknown, multiple factors are responsible for the abnormality in cellular development. A solid malignant tumor is an abnormal mass of tissue that usually does not contain cysts or liquid areas. These solid tumors vary according to their site of origin, for instance, sarcomas (tumors arising from connective tissue), carcinomas (tumors arising from epithelial tissue), and lymphomas (tumors arising from the lymphatic system). However, the term solid tumor is used to distinguish between a localized mass of tissue and leukemia [1]. The biology of cancer is a complex interplay of many underlying processes, taking place at different scales both in space and time.

Cancer remains a leading cause of death across the globe and approximately 10 million deaths have been reported due to cancer or related complications in 2020 [2,3]. The most common cancers in both the developed and developing world are breast, lung, colon, rectum, and prostate cancers [2,3]. The prevalence rates of the leading cancers over five years reveal that the most common forms of cancers in adult males are lung cancer (13.1%), lip and oral cavity cancer (11.9%), other pharynx cancer (8.2%), colorectal cancer (6.5%), stomach cancer (4.7%), esophageal cancer (4.1%), non-Hodgkin's lymphoma (4.7%), Hodgkin's lymphoma (2.2%), bladder cancer (3.4%), and prostate cancer (2.3%) [4,5].

Additionally, the most common cancers in adult females are breast cancer (32.8%), cervical cancer (26.1%), lip and oral cavity cancer (6.5%), ovarian cancer (3.3%), colorectal cancer (2.7%), lung cancer (2.0%), esophageal cancer (1.9%), stomach cancer (1.8%), non-Hodgkin's lymphoma (1.3%), and Hodgkin's lymphoma (0.8%) [4,5]. Some other malignancies such as thyroid cancer and renal cell carcinoma are also common. Besides, entities such as malignant melanoma, osteosarcoma, chondrosarcoma, soft tissue sarcoma, testicular cancer, vaginal cancer, vulvar cancer, and mesothelioma are regarded as rare cancers.

There are 1.3 million to 1.5 million cancer patients in Bangladesh and about 0.2 million patients with newly diagnosed cancer each year [4,5]. The etiology of cancer is multifactorial, and the disease process varies according to its site of origin. Tobacco is the single most identified risk factor for cancer. A host of other environmental exposures, certain infections, and genetic predispositions play an important role in carcinogenesis.

Traditionally, it is widely perceived that cancer affects the elderly population [6-8]. However, numerous studies show that cancer cases have been increasing in adults less than 50 years of age in most parts of the world [6-8]. The increase in the number of cancer cases, especially early-onset cancer, has substantial social, personal, as well as economic aspects [9]. The increasing trend of cancer incidence has compelled public health officials and the medical community to exert more effort in cancer prevention and treatments.

## Materials and methods

This observational cross-sectional study was performed at a medical teaching hospital in central Bangladesh. The study subjects were enrolled after obtaining institutional ethical clearance (Ethical Committee of Dhaka Medical College Hospital - Post Graduate Research Memo No.: MEU-DMC/ECC/2018/43; dated: March 18, 2018) and written informed consent from all study participants. All patients (age > 18 years) with a histopathologically confirmed solid malignant tumor in the Department of Medicine of Dhaka Medical College Hospital, Dhaka, Bangladesh between January 2018 and June 2018 were included in this study. Critically ill patients requiring intensive care unit (ICU) support, and other malignancies such as multiple myeloma and leukemia were excluded from this study. Complete sociodemographic characteristics, risk factors, clinical presentation, and histopathological characteristics of the tumor were recorded in the study. A malignant solid tumor was diagnosed in the patient consistent with compatible clinical presentation of solid malignancy and further confirmation by biopsy followed by histopathological examination was done to diagnose the appropriate tumor type.

The sample size was calculated using G*Power version 3.1.9.4 where the mean difference between two independent means (two groups) was applied taking effect size as 0.50, alpha error as 0.05, and power as 80% with location ratio as 1. The total sample size was 100. All statistical analyses were performed using the Statistical Package for the Social Sciences (SPSS) program, version 16 for Windows (IBM Corp., Armonk, NY). Continuous variables were expressed as mean ± SD and categorical variables as frequency and percentage. Comparison of the mean (continuous variables) was done by Student’s t-test. The association between categorical variables was assessed by the chi-square test. Correlation analyses were done by Pearson's correlation coefficient for continuous variables. The significance of the result as determined by a 95% confidence interval and a p-value < 0.05 was considered to be statistically significant.

## Results

A total of 100 histopathologically confirmed diagnosed patients with malignant solid tumors were included in this study. Among them, 59% were males and 41% were females. The mean age of the study participants was 47.5 years (SD: ±4.20), and most of them (27%) were within the 58-67 years age group. Of the participants, 48% completed their primary education. Most of the females (33%) were housewives. Among the males, 22% were businessmen, 17% were service holders, 16% were farmers, and 12% were either involved in other occupations or retired. Of the participants, 59% hailed from the middle socio-economic class and 31% belonged to the lower socio-economic class. A total of 51% were sedentary workers. Moreover, 12% were smokers and 11% were ex-smokers (Table 1).

**Table 1:** Distribution of the socio-demographic characteristic (n = 100)

Characteristics	Group	Frequency	Percentage (%)
Age (years)	18-27	7	7
	28-37	10	10.0
	38-47	26	26.0
	48-57	19	19.0
	58-67	27	27.0
	68-77	11	11.0
Sex	Male	59	59.0
	Female	41	41.0
Educational status	Illiterate	15	15.0
	Primary education	48	48.0
	Secondary education	30	30.0
	Higher secondary	6	6.0
	Graduation	1	1.0
Occupational status	Service holder	17	17.0
	Farmer	16	16.0
	Businessmen	22	22.0
	Housewife	33	33.0
	Others/retired	12	12.0
Socio-economic status	Upper class	10	10.0
	Middle class	59	59.0
	Lower class	31	31.0
Marital status	Married	94	94.0
	Unmarried	6	6.0
Lifestyle	Sedentary	51	51.0
	Hard worker	49	49.0
Personal habit	Smoker	12	12.0
	Ex-smoker	11	11.0
	Nonsmoker	61	61.0
	Betel nut chewer	7	7.0

**Figure 1:**
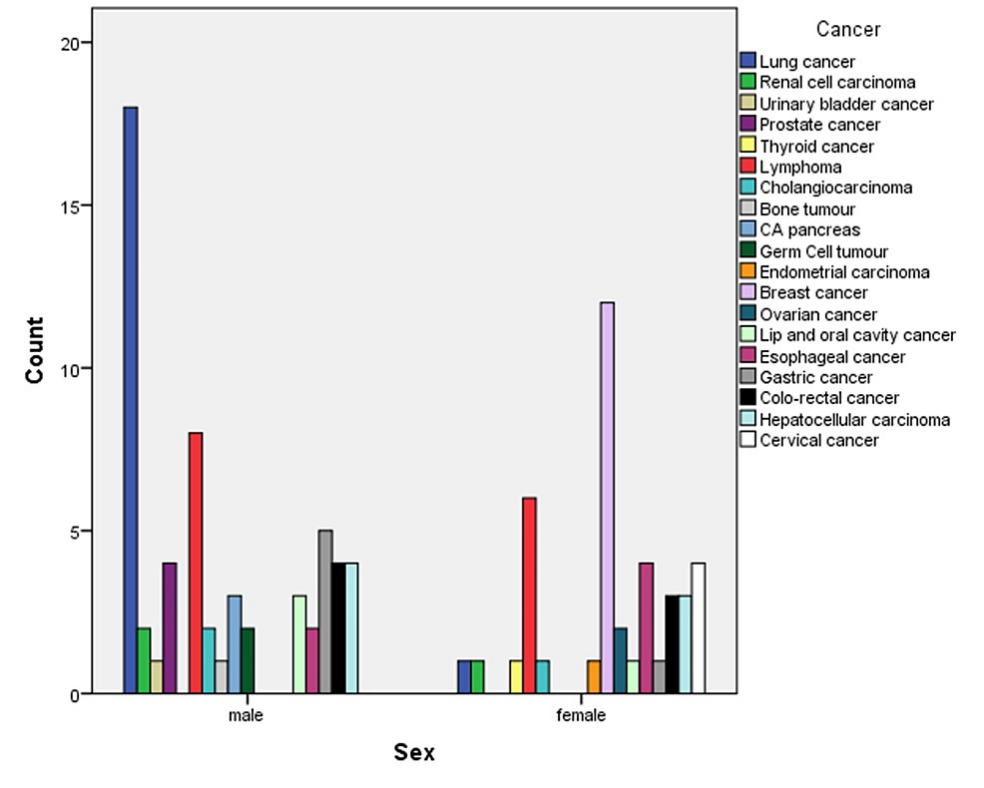
Distribution of solid malignant tumors among adult males and females CA: carcinoma.

Among the respondents, 19% had lung cancer, 14% had lymphoma, 29.27% had breast cancer, 7% had colorectal cancer, 7% had hepatocellular carcinoma, 6% had esophageal cancer, 6% had gastric cancer, 4% had lip and oral cavity cancer, 9.76% had cervical cancer, 6.78% had prostate cancer, 3% had cholangiocarcinoma, 3% had pancreatic cancer, 3% had renal cell carcinoma, and 4.88% had ovarian cancer (Figure 1).

Among the lung cancer patients, 94.7% were males with a mean age of 57.5 years (SD: ±7.09). Out of the lung cancer patients, 47.4% were smokers. The most common clinical presentation was found to be chronic cough (89.47%), hemoptysis (52.63%), respiratory distress (47.37%), weight loss (73.68%), mass lesion (47.37%), pleural effusion (15.79%), superior vena cava obstruction (SVCO) syndrome (10.53%), collapse (5.3%), and anemia (26.31%). Adenocarcinoma (36.8%) was the most common histopathological type followed by squamous cell carcinoma (31.6%) and small cell lung cancer (26.3%) (Table 2).

**Table 2:** Characteristics of lung cancer patients (n = 19) SVCO: superior vena cava obstruction.

Characteristics	Group	Frequency	Percentage (%)
Symptoms	Chronic cough	17	89.47
	Hemoptysis	10	52.63
	Respiratory distress	09	47.37
	Weight loss	14	73.68
Signs	Anemia	05	26.31
	Pleural effusion	03	15.79
	Mass lesion	09	47.37
	Collapse	01	5.3
	SVCO syndrome	02	10.53
Histopathological types	Adenocarcinoma	07	36.8
	Squamous cell carcinoma	06	31.6
	Small cell lung cancer	05	26.3
	Undifferentiated lung cancer	1	5.3
Personal habit	Smoker	09	47.4
	Ex-smoker	03	15.8
	Nonsmoker	06	31.6
	Betel nut chewer	02	10.52
Sex	Male	18	94.7
	Female	01	5.3
Age (years)	38-47	01	5.3
	48-57	05	26.3
	58-67	08	42.1
	68-77	05	26.3

All the breast cancer patients were females with a mean age of 42.5 years (SD: ±9.53). The most common clinical presentation was found to be breast lump (91.67%), axillary node (66.67%), mastalgia (16.67%), and nipple discharge (8.3%). Infiltrating ductal carcinoma was the most common histopathological type constituting almost 100% of all the cases. Besides, the right breast (58.33%) was found to be more involved than the left breast (Table 3).

**Table 3:** Characteristics of breast cancer patients (n = 12)

Characteristics	Group	Frequency	Percentage (%)
Symptoms	Breast lump	11	91.67
	Mastalgia	02	16.67
	Nipple discharge	01	8.3
	Axillary nodes	08	66.67
Signs	Anemia	05	41.67
	Breast mass	11	91.67
	Axillary lymphadenopathy	06	50.0
	Ulcerative lesion	02	16.67
Histopathological type	Infiltrating ductal cell carcinoma (IDC)	12	100.0
Sites of involvement	Right breast	07	58.33
	Left breast	05	41.67
Sex	Male	0	0
	Female	12	100
Age (years)	18-27	01	8.3
	28-37	02	16.67
	38-47	05	41.67
	48-57	02	16.67
	58-67	02	16.67

Among the patients suffering from oral cavity cancer, 75% were males with a mean age of 42.5 years (SD: ±18.26). Among them, 50% were smokers and 25% chewed betel nuts. The most common presentation of oral cavity cancer was ulcer (50%), anemia (50%), and cervical lymphadenopathy (50%).

Among the esophageal cancer patients, 66.67% were females having a mean age of 57.5 years (SD: ±9.13). The most common presentation of esophageal cancer included progressive dysphagia (100%), weight loss (83.33%), and anemia (100%). The most common site of involvement in esophageal cancer was the lower third (66.67%) of the esophagus. The most common histopathological type was adenocarcinoma (66.67%) followed by squamous cell carcinoma (33.33%).

Among the study subjects of gastric cancer, 83.33% were males with a mean age of 59.17 years (SD: ±9.66). The most common presentation of gastric cancer was weight loss (83.33%), dyspepsia (66.67%), early satiety (50%), vomiting (50%), anemia (100%), epigastric mass (50%), and enlarged Virchow's gland (33.33%). Histologically, intestinal-type adenocarcinoma constituted 83.33% of all gastric cancers and the rest were found to be diffuse gastric-type adenocarcinoma.

The mean age of colorectal cancer patients was 52.5 years (SD: ±10.80) and out of them, 57.14% were males. Common presentations of colorectal cancer were alteration of bowel habits (85.71%), per-rectal bleeding (71.13%), anemia (71.43%), hepatomegaly (42.86%), and ascites (28.57%). The most common sites of involvement in colorectal cancer were the rectum (42.86%) and sigmoid colon (28.57%). Adenocarcinoma (100%) was found to be the most common histopathological type.

The mean age of hepatocellular carcinoma patients was 52.5 years (SD: ±5.77) and out of them, 57.14% were males. Furthermore, the most common clinical presentation of hepatocellular carcinoma was found to be abdominal pain (100%), abdominal distension (85.71%), jaundice (57.14%), anemia (100%), hepatomegaly (85.71%), and ascites (42.86%). Alpha-fetoprotein was found to be raised in 57.14% of the cases of hepatocellular carcinoma. Moreover, 57.14% had hepatocellular carcinoma related to hepatitis B viral infection and 14.29% had hepatocellular carcinoma related to hepatitis C viral infection.

Males (57.14%) were predominant among the lymphoma patients with a mean age of 42.5 years (SD: ±8.77). The most common clinical presentation of lymphoma was found to be generalized lymphadenopathy (71.43%). Additionally, 21.43% of the lymphoma patients had cervical lymphadenopathy. Non-Hodgkin's lymphoma (71.43%) was the most common histopathological type (Table 4).

**Table 4:** Characteristics of lymphoma patients (n = 14)

Characteristics	Groups	Frequency	Percentage (%)
Symptoms	Swelling in the cervical region	03	21.43
	Swelling in multiple sites of the body	10	71.43
	Fever	08	57.15
	Weight loss	06	42.86
	Night sweat	04	28.57
	Itching	04	28.57
Sign	Only cervical lymphadenopathy	03	21.43
	Generalized lymphadenopathy	10	71.43
	Hepatosplenomegaly	04	28.57
	Anemia	02	14.28
Histopathological type	Non-Hodgkin's lymphoma	10	71.43
	Hodgkin's lymphoma	04	28.57
Sex	Male	08	57.14
	Female	06	42.86
Age (years)	18-27	02	14.28
	28-37	05	35.71
	38-47	04	28.57
	48-57	02	14.28
	58-67	01	7.14

The mean age of carcinoma prostate patients was 67.5 years (SD: ±4.08). The most common clinical presentation of carcinoma prostate was found to be hematuria (100%), lower urinary tract symptoms (LUTS) (75%), and anemia (100%). Out of all the carcinoma prostate patients, 75% had hard, irregular prostate on digital rectal examination (DRE). The mean age of cervical cancer patients was 37.5 years (SD: ±4.082). The most common clinical presentation of cervical cancer was found to be per vaginal (PV) discharge (100%), PV bleeding (75%), edema (50%), and ascites (25%). All the cervical cancer patients had friable ulcerative growth on PV examination. Squamous cell carcinoma was the most common histopathological type of cervical cancer. The mean age of renal cell carcinoma (RCC) patients was 47.5 years (SD: ±5.0). Among them, 66.67% were males. The most common clinical presentation of RCC was found to be hematuria (100%) and loin pain (66.67%). The right kidney (66.67%) was mostly involved. Furthermore, clear cell variety was the most common histopathological type.

## Discussion

Most of our findings match what others have found regarding prevalence rates. Uddin et al. and Noronha et al. had similar findings to our study, where prevalence rates of the leading cancers over the last five years were similar to this study, with lung cancer being the most common cancer among adult males and breast cancer being the most common cancer among females [4,5]. In a previous study done by Singh and Rohtagi (2017), the most common presentation of lung cancer was chronic cough with a mean age of 57.5 years [10]. Most of their study participants belonged to the 58-67 years age group [10]. There was male predominance and association with smoking [10]. Adenocarcinoma was found to be the most common histopathological subtype followed by squamous cell carcinoma [10].

A common presentation of breast cancer in our study was the presence of a breast lump with a mean age of 42.5 years. We found a female predominance. The right breast was the most involved and infiltrating ductal carcinoma was the histopathological type, which correlates with the findings from Takalkar et al.'s study [11].

The most common presentation of lip and oral cavity cancer in our study was the presence of an ulcer with swelling. We found a male predominance. The most common sites of involvement were the lip and buccal mucosa, followed by the tongue. Our study illustrates that most of the Bangladeshi people who developed this type of cancer were habitual smokers and betel nut chewers.

The most common presentation of esophageal cancer in our study was progressive dysphagia. The lower third of the esophagus was most involved. And adenocarcinoma was found to be the most common histopathological subtype, which is not comparable with Gupta et al.’s study since Gupta et al. found that the upper and middle third of the esophagus was most involved and squamous cell carcinoma was the most common histopathological subtype [12].

Gastric cancer is the most common cancer in the world and males are most involved. The most common symptoms of gastric cancer in our study were weight loss, dyspepsia, abdominal pain, early satiety, and vomiting. We found that the most common histopathological subtype was intestinal-type adenocarcinoma, which resembles the findings of a previous study done by Sushma et al. [13].

Colorectal cancer ranks second in cancer-related deaths and third among all cancers worldwide [14]. Our study showed that males are most affected by colorectal cancer. The most common symptoms of colorectal cancer were alteration of bowel habits, per rectal bleeding, weight loss, abdominal mass, and tenesmus. The most common site of involvement was the rectum and adenocarcinoma was found to be the most common histopathological subtype.

In our study, most of the patients suffering from hepatocellular carcinoma had raised alpha-fetoprotein, which corresponds to Gani et al.'s study [15]. Hepatocellular carcinoma is a major health problem with a high incidence and mortality all over the world. In our study, the most common clinical presentations were abdominal pain, ascites, and jaundice. We found a male predominance and most of the patients were infected with the hepatitis B virus.

Lymphoma is a disease of the lymphoid organs so it can occur in any part of the body. Kusminsky et al. found that the most common presentations of lymphoma were generalized lymphadenopathy, cervical lymphadenopathy, hepatosplenomegaly, anemia, fever, and weight loss [16]. Males were more affected by lymphoma than females. Non-Hodgkin's lymphoma was revealed to be the most common histopathological type followed by Hodgkin's lymphoma; both types have a good prognosis and respond better to treatment. In this regard, Kusminsky et al.’s findings correspond to the findings in our study.

Zeigler-Johnson et al. have similar findings to ours with prostate cancer [17]. Prostate cancer is the second most diagnosed cancer in men worldwide. In our study, the most common presentations of prostate cancer were LUTS, hematuria, bone pain, urinary retention, and anemia. Hard irregular prostate on DRE was also another common finding. Adenocarcinoma was the most common histopathological subtype. Our study revealed that prostate cancer usually occurs in elderly patients, which resembles the findings of a previous study done by Zeigler-Johnson et al. [17]. However, Zeigler-Johnson et al. found differences in the presentation of prostate cancer [17]. Men in developing countries present with more advanced diseases compared to men in the US.

Cervical cancer is the leading cancer causing death among women in developing countries. It usually occurs in women who are of lower socioeconomic status. In our study, the most common presentations of cervical cancer were per vaginal foul-smelling watery discharge, per vaginal bleeding, post-coital bleeding, and constipation. The presence of friable ulcerative growth per vaginal examination was another common finding. In our study, some patients presented with features of metastasis such as ascites and obstructive uropathy. Ectocervix was the most common site of involvement and squamous cell carcinoma was revealed to be the most common histopathological subtype, which correlates with a previous study done by Umate et al. [18]. In our study, the most common presentations of RCC were hematuria and loin pain. We found that the right kidney was the most common site of involvement for RCC. Clear cell variety was the most common histopathological subtype, which correlates with a previous study done by Padmanabhan et al. [19].

In our study, the patients with pancreatic cancer primarily presented with features of metastasis. The most common presentations of pancreatic cancer were epigastric lump, ascites, and jaundice. We found that the tumor marker carcinoembryonic antigen-19.9 (CA-19.9) was very high in almost all patients and adenocarcinoma was the most common histopathological subtype. Among our study participants, 4.88% were patients with ovarian carcinoma and most of them were between 38 and 47 years of age. The most common symptom of ovarian carcinoma was dyspepsia. Our study delineates that endometrioid adenocarcinoma comprised 2% of the cases. However, a small study population may not reflect the actual scenario. Among our study respondents, 2.44% of patients had endometrial carcinoma. They were within 38-47 years of age and menorrhagia was the common symptom.

In this study, there were two cases (2%) of germ cell tumors among male patients and they were within 18-27 years of age. The most common symptoms were testicular swelling and the presence of abdominal mass. Seminoma was the most common histopathological subtype, which corresponds to another study done by Komatsubara and Carvajal (2016) [20]. In our study, 1% of patients had osteosarcoma and they were between 18 and 27 years of age. The knee joint was the most common site of involvement in osteosarcoma. The most common symptoms of osteosarcoma were painful swelling and restriction of movement of the knee joint, which correlates with Komatsubara and Carvajal's (2016) study [20].

In our study, bladder tumor was found to be the most common in industrial areas and its incidence increased with exposure to cigarettes and arylamines. The most common presentations of bladder tumors were hematuria and urinary retention. And transitional cell carcinoma was the most common histopathological subtype found in this study.

Thyroid cancer is a more predominant malignancy in women than in men, which is likely due to the more variable hormonal environments in women than in men. In this study, 1% of patients had thyroid carcinoma. They were 48-57 years of age. We found that females are more affected by thyroid carcinoma than males. Our study depicts that the most common presentation of thyroid carcinoma was the presence of solitary thyroid swelling. Papillary carcinoma was found to be the histopathological subtype, which corresponds to another study done by Girardi et al. [21].

Limitations of the study

During our study period, we faced various types of limitations, which may influence the external or internal validity of this study. The study was conducted with a semi-structured interviewer-administered questionnaire. This study included patients admitted to the hospital with severe symptoms and when the data were collected, the patients were undergoing medical treatment. Due to the ongoing treatment, the findings which should be present were absent to some extent. The relatively small subsample of illiterate patients provided limited power to reject risk differences with small magnitude. Since the study was conducted at a public hospital in Dhaka city and the respondents of the research work were self-selected purposively, hence, it cannot be assumed that this sample could be representative of the entire population of Bangladesh. The results may vary across different social-demographic or cultural situations.

## Conclusions

Our small hospital-based study describes the socio-demographic status of patients with solid malignant tumors and the clinicopathological presentation among adult patients. Our research reflects that the solid malignant tumor is not only a disease of the people of the higher socio-economic class who usually lead sedentary lifestyles but also may affect individuals from the relatively lower socio-economic classes. Bangladesh is considered a least developed country by the United Nations (UN), but, as a relatively young country, it is rapidly reducing poverty. Further research including a national survey and incorporation of the broad-scale impacts of poverty within the country of Bangladesh is warranted.
